# Exogenous Melatonin Alleviates Oxidative Damages and Protects Photosystem II in Maize Seedlings Under Drought Stress

**DOI:** 10.3389/fpls.2019.00677

**Published:** 2019-05-24

**Authors:** Bo Huang, Yang-Er Chen, Yu-Qing Zhao, Chun-Bang Ding, Jin-Qiu Liao, Chao Hu, Li-Jun Zhou, Zhong-Wei Zhang, Shu Yuan, Ming Yuan

**Affiliations:** ^1^College of Life Sciences, Sichuan Agricultural University, Ya’an, China; ^2^College of Resources Science and Technology, Sichuan Agricultural University, Chengdu, China

**Keywords:** melatonin, maize, drought, antioxidant system, photosynthesis

## Abstract

The protective role of melatonin in plants against various abiotic stresses have been widely demonstrated, but poorly explored in organ-specific responses and the transmission of melatonin signals across organs. In this study, the effects of melatonin with the root-irrigation method and the leaf-spraying method on the antioxidant system and photosynthetic machinery in maize seedlings under drought stress were investigated. The results showed that drought stress led to the rise in reactive oxygen species (ROS), severe cell death, and degradation of D1 protein, which were mitigated by the melatonin application. The application of melatonin improved the photosynthetic activities and alleviated the oxidative damages of maize seedlings under the drought stress. Compared with the leaf-spraying method, the root-irrigation method was more effective on enhancing drought tolerance. Moreover, maize seedlings made organ-specific physiological responses to the drought stress, and the physiological effects of melatonin varied with the dosage, application methods and plant organs. The signals of exogenous melatonin received by roots could affect the stress responses of leaves, and the melatonin signals perceived by leaves also led to changes in physiological metabolisms in roots under the stress. Consequently, the whole seedlings coordinated the different parts and made a systemic acclimation against the drought stress. Melatonin as a protective agent against abiotic stresses has a potential application prospect in the agricultural industry.

## Introduction

Water is thought to be one of the main environmental elements which restrict crop growth, development and yield. Water deficit in the soil, described as drought stress, usually results in various physiological and metabolic disorders of plants. Drought stress is the key restrictive factor for crop production, and has led to a rough 40% reduction of maize over the past 25 years in the world (Daryanto et al., 2016). Responses of plants to drought are usually interconnected and multiple. A lot of researches have shown that drought leads to limitation in total nutrient uptake, decreases in water and photosynthetic pigment contents, depression of the photochemical efficiency, and reduction in growth (Wahid and Rasul, 2005; Farooq et al., 2009; Chen et al., 2016a). Most of these drought damages are involved in the photosynthetic process in plants. Some researchers have demonstrated that the reduction of the photosynthetic activity might be due to non-stomatal and stomatal limitations under drought stress (Zlatev and Yordanov, 2004). In addition, several reports have indicated that drought stress led to damages to the oxygen evolving complex and the reaction center of photosystem II (PSII; Lu and Zhang, 1999).

Furthermore, a major effect of drought in plant leaves and roots is the rapid accumulation of reactive oxygen species (ROS), which can cause photoinhibition, the peroxidation of membrane lipids, degradation of biomacromolecules, and oxidative damages (Farooq et al., 2009; Chen et al., 2016b). Under drought stress, excessive ROS can impair the chloroplasts, decrease the photochemical reactions, and finally suppress the photosynthesis and yield of the crop (Li T. et al., 2014). Plants have developed several enzymatic and non-enzymatic antioxidant protection mechanisms to counteract the damaging effects of ROS under environmental stresses (Wang et al., 2010). At the same time, plants can deal with environmental stresses through increasing dissipation of excess excitation energy or synthesizing and accumulating some stress-resistant substances (Chen et al., 2017). During the last 50 years, global climate change has significantly adverse effects on crop yields because of the increases in drought and heat stresses (Hillel and Rosenzweig, 2002). Therefore, the improvement of crop yields under various abiotic stresses is an urgent task to meet the booming food demands of the ever increasing population. The application of exogenous plant growth substances has been demonstrated to be an effective approach to enhance crops resistance to drought (Peleg and Blumwald, 2011).

Melatonin (N-acetyl-5-methoxytryptamine) was initially identified in the bovine pineal gland (Lerner et al., 1958). It acts as a biological regulator of circadian rhythms, sleep, immunological systems, sexual behavior, reproduction, and antioxidative activities in animals (Hattori et al., 1995; Jan et al., 2009; Reiter et al., 2010; Hardeland et al., 2012; Carrillo-Vico et al., 2013). In addition, melatonin as a naturally antioxidant may play important regulatory roles in the growth and development, and various stress responses in plants (Dubbels et al., 1995; García et al., 2014). Many evidences have demonstrated that melatonin can enhance the resistance against multiple adverse environmental factors, such as cold, drought, salinity, high temperature, ultraviolet radiations, heavy metals, and pathogen infections (Afreen et al., 2006; Posmyk et al., 2008; Yin et al., 2013; Han et al., 2017). Although the antioxidative effects of melatonin on alleviating different environmental stresses have been widely investigated in plants (Wang et al., 2013b; Zhang et al., 2013; Li C. et al., 2014; Ye et al., 2016), there is little study on organ-specific physiological responses to melatonin applications under drought stress.

As one of the three primary crops across the world, maize (*Zea mays* L.) is drought-sensitive. In recent decades, drought has found to be the largest limitation for the production of maize in many regions of the world. Therefore, it is of important significance to increase the drought resistance of maize and make it maintain productivity under drought. Although many reports have shown that the application of exogenous melatonin can enhance drought tolerance in different plant species, there is no study on the comparison of photosynthesis and oxidative damages under drought stress through the two different methods of melatonin applications. Furthermore, few researches have looked into the role of melatonin in protecting the photosynthetic apparatus, and the organ-specific physiological responses to drought stress have not been reported previously. In this research, we investigated the protective role of melatonin in maize roots and leaves under drought stress with two different application methods through comparing ROS accumulation, the abilities of antioxidant enzymes, and photochemical capacities. Here we expect to provide the better understanding in the regulatory role and effective application methods of melatonin in improving of the drought tolerance of plants.

## Materials and Methods

### Plant Materials and Treatments

Maize (*Z. mays* L.) seeds were sterilized with 0.1% HgCl_2_ (w/v) for 10 min and washed with distilled water for 5 times. These seeds germinated on the wetted filter paper at 25°C for 1 day in dark. The seedlings with uniform size were transplanted in black plastic pots with equal quantity nutritive soil and placed in intelligent greenhouse, which was set to a photoperiodic cycle (14 h light, 10 h dark at 25 and 20°C, respectively), roughly 70% relative humidity, and 180 μmol m^-2^ s^-1^ light intensity. Each pot (14 cm diameter at bottom and 16 cm high) contained six seedlings, and irrigated with 1/2 Hoagland’s solution every 2 days.

One-week-old maize plants with the same size were selected and treated with two different application methods for 2 days. One method was that the maize seedlings were irrigated with different concentrations of melatonin solutions (I). The other method was that melatonin solution was sprayed on the leaves 5 times every day at 2 h intervals with the same concentrations containing 0.05% (V/V) Tween-20 as a non-ionic surfactant (II). Then, all the plants were divided into nine groups as follows: (1) non-melatonin plus well watered (Control, CK); (2) 20 μmol/L melatonin plus well watered (M_20_); (3) 100 μmol/L melatonin plus well watered (M_100_); (4) 0 μmol/L melatonin plus progressive drought stress (Dr); (5) 20 μmol/L melatonin pretreatment plus progressive drought stress; (6) 100 μmol/L melatonin pretreatment plus progressive drought stress. (7) 0.05% Tween-20 solution was sprayed on the leaves and progressive drought stress; (8) 0.05% Tween-20 solution plus 20 μmol/L melatonin pretreatment and progressive drought stress; (9) 0.05% Tween-20 solution plus 100 μmol/L melatonin pretreatment and progressive drought stress. Then stopped watering to apply progressive drought stress. After drought for 7 days, rehydration was applied to all drought treatment groups. Root and the second leaves from the top were gathered at the final day of drought treatment for the following measurements.

### Determination of Chlorophyll Content, Leaf Relative Water Content (RWC), Plant Dry Weight and Soil Water Content

Fresh leaves of 1 cm^2^ were thoroughly cut and submerged into 2 mL 80% acetone in the dark for 24 h (4°C). The absorbance of the solution was measured at 646.6 nm and 663.6 nm with a spectrophotometer *A560* (AOE Instruments, Shanghai, China). Chlorophyll content was calculated according to Porra et al. (1989). The leaf relative water content (RWC) and the plant dry weight were determined according to Li T. et al. (2014). Soil water content was measured by oven drying soil at 110°C for 24 h (Sainju et al., 2017).

### Melatonin Extraction and Quantification

The measurement of melatonin was done with the method of Sturtz et al. (2011). 1 g of fresh tissue samples were grounded and 2 mL of acetone was added, then transferred into 10 mL tube. After mixing well and shaking with ultrasonic bath for 0.5 h at 25°C, the mixture was centrifuged with 3000 *g* at 4°C, the supernatant was evaporated under vacuum and resolved with 2 mL water. After purification with C18-SPE cartridges (Bonna-Agela Technologies Instruments, Tianjin, China), the quantitative analysis of melatonin was carried out through a high performance liquid chromatography (HPLC) system (1290 LC, Agilent, Santa Clara, CA, United States) with a tandem mass spectrum (MS) system (6470 LC-MS/MS, Agilent, Santa Clara, CA, United States). LC-MS/MS parameters were set as Han et al. (2017).

### Determination of Photosynthetic Parameters and Chlorophyll Fluorescence

A portable gas exchange system GFS-3000 (Heinz Walz Instruments, Effeltrich, Germany) was used to measure photosynthetic parameters according to Kaling et al. (2015). The measurement was performed at ten o’clock in the morning on the 2nd leaf from the top. A 4 cm^2^ area of the leaf was clamped and exposed to the general parameter (flow of 750 μmol s^-1^, leaf temperature of 25°C, incident light intensity of 180 μ mol m^-2^ s^-1^ and a relative humidity of 70%). Photosynthesis and gas exchange rates were recorded after reached a steady state. Water-use efficiency (WUE) was calculated as the ratio of net photosynthetic rate to transpiration rate.

Chlorophyll fluorescence was imaged with a modulated imaging fluorometer (the Imaging PAM M-Series Chlorophyll Fluorescence System, Heinz Walz Instruments, Effeltrich, Germany). The maximum efficiency of PSII photochemistry (F_v_/F_m_), the quantum yield of PSII photochemistry [Y(II)], photochemical quenching (qP) and non-photochemical quenching (NPQ) were imaged and calculated after adaption in the dark for 30 min (Naranjo et al., 2016).

### Quantification of Lipid Peroxidation and Relative Electrolyte Leakage

Taking malondialdehyde (MDA) content as assessment index of lipid peroxidation, and MDA content was determined with thiobarbituric acid (TBA) method via recording the absorbance at 600, 532, 450 nm as described by Zhang et al. (2013). The electrolyte leakage (EL) of the root and leaves was determined based on Fan et al. (2015). 0.5 g of detached material was washed with ultrapure water, and cut into 0.5 cm fragments. Then, the samples were submerged into 30 mL deionized water for 24 h, and the conductivity (EL1) was recorded with a conductance meter DDS-309+ (ARK Instruments, Chengdu, China). Then the sample in the tube were completely damaged by boiling water bath for 30 min, and the conductivity (EL2) was read again after cooling to room temperature. Relative electrolyte leakage (REL) was calculated as the ratio of EL1 to EL2.

### Quantification of Soluble Sugar Contents and Proline

The soluble sugar content was performed with the anthrone method as described by Shi et al. (2015b). Briefly, 0.1 g sample was added to 2 mL of 80% (V/V) ethanol at 80°C for 30 min. 100 μL of extracts was mixed with 2 mL anthrone, and then boiled for 10 min. The absorption was recorded at 630 nm, and the content was calculated according to the calibration curve of sucrose standard. Proline was determined based on Ye et al. (2015). Briefly, 0.5 g frozen powder sample was blended with 5 mL of 3% (W/V) sulfosalicylic acid. Then, mixed with 2 mL of ninhydrin reagent and 2 mL of glacial acetic acid, and heated in boiling water for 30 min. After cooling to 25°C, the reaction solution was centrifugation at 10000 *g* for 10 min, and the absorbance at 520 nm was recorded and the proline level was calculated according to the standard curve.

### Assays for Cell Death

Dead cells were stained by trypan blue (Lin et al., 2012). The leaves were detached and infiltrated with the lactophenol-trypan blue solution for 1 h at 70°C, then placed in the boiling water for 5 min and stained for 12 h. The background staining was removed by 2.5 g/mL chloral hydrate solution for 3 days, tissues were equilibrated with 70% glycerol for photographing.

### Measurements of Reactive Oxygen Species (ROS)

Histochemical detection of ROS was conducted as described by Chen et al. (2017). Briefly, hydrogen peroxide (H_2_O_2_) and superoxide anion radicals (O_2_^-^) were visually detected with 0.5 mg/mL 3, 3- diaminobenzidine (DAB) and 1 mg/mL nitro blue tetrazolium (NBT), respectively. Then, the tissues were decolorized for 2 h in boiling ethanol (85%). The quantification of H_2_O_2_ and superoxide anion radicals was determined as described by Chen et al. (2016a).

### Determination of Antioxidant Enzyme Activities

Fresh tissue of 0.2 g was grounded in 4 mL of 150 mM, pH 7.8 ice-cold sodium phosphate buffer on ice, and then centrifuged at 12000 *g* at 4°C for 20 min. The supernatant was used for the next enzyme activity assays.

Superoxide dismutase (SOD) activity was evaluated according to the ability to inhibit the photochemical reduction of NBT (Abedi and Pakniyat, 2010). Guaiacol peroxidase (POD) activity was evaluated with the ability to convert guaiacol to tetraguaiacol (Abedi and Pakniyat, 2010). Catalase (CAT) activity was assayed by monitoring the absorbance of H_2_O_2_ at 240 nm (Wang, 1995). Ascorbate peroxidase (APX) activity was measured based on the decrease in ascorbate (Nakano and Asada, 1981). Glutathione peroxidase (GPX) activity was estimated as described by Sayfzadeh and Rashidi (2010). Glutathione reductase (GR) activity was measured based on Balabusta et al. (2016) and depends on the rate of the oxidation of NADPH. Activity of dehydroascorbate reductase (DHAR) was determined by the increase in reduced ascorbate (de Pinto et al., 2000).

### Determination of Antioxidant Metabolites

The antioxidant metabolites include reduced ascorbic acid (AsA), dehydroascorbate (DHA), reduced glutathione (GSH) and oxidized glutathione (GSSG). They were determined with the enzymatic cycling assay method (Wang et al., 2013b).

### Isolation of Thylakoid Membranes and Western Blotting

The thylakoid membrane protein was prepared as described by Fristedt et al. (2010). Thylakoid membrane protein was quantified based on total chlorophyll (Porra et al., 1989). For analysis of thylakoid proteins, an equivalent chlorophyll basis were loaded in gels and were separated by 15% sodium dodecyl sulfate polyacrylamide gel electrophoresis. Then, the protein was transferred to a PVDF membrane (Bio-Rad, Hercules, CA, United States). The primary antibodies including anti-D1, anti-D2, anti-LHCB1, anti-LHCB2, and anti-LHCB3 were purchased from *Agrisera* (Umea, Sweden). A chemiluminescent detection system (ECL, GE Healthcare, Buchinghamshire, United Kingdom) was used to perform the immunoblots. The quantification of immunoblots was done with the Quantity One software (Bio-Rad, Hercules, CA, United States).

### Statistical Analysis

We repeated all experiments at least three times of independent experiments. Results were analyzed via one-way ANOVA and then Duncan’s multiple range test was carried out to indicate a significant difference at *P*<0.05. All data were expressed as means ± standard deviation (SD).

## Results

### The Effects of Exogenous Melatonin on Plant Growth

**FIGURE 1:**
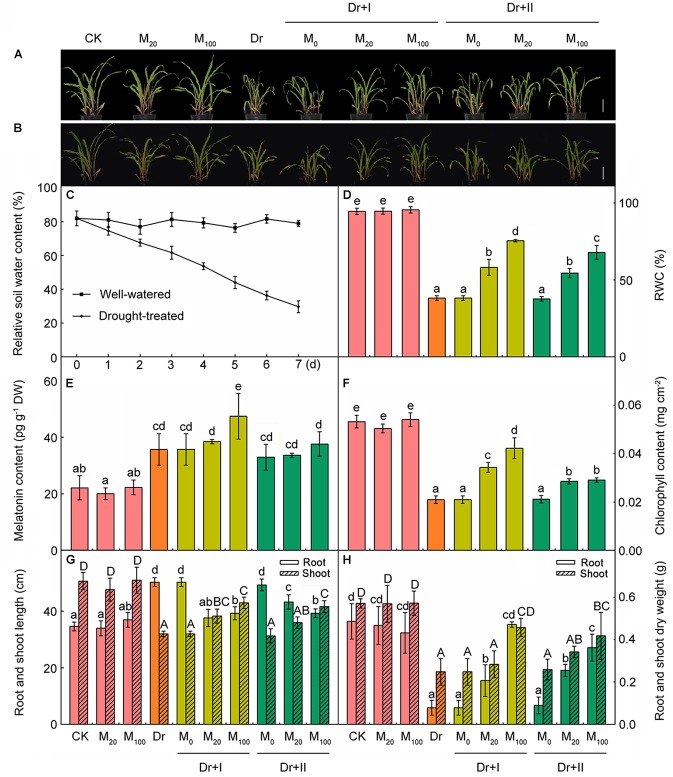
Exogenous melatonin improved drought stress resistance in maize seedlings. **(A)** The phenotype of melatonin pre-treated or non-treated seedlings after 7-day drought stress; **(B)** the phenotype of plants after 24 h rehydration; **(C)** the soil water content during drought stress; **(D)** leaf relative water content after 7-day drought; **(E)** the content of melatonin in leaves after 7-day drought; **(F)** the chlorophyll content in leaves after 7-days drought stress; **(G)** the length of root and shoot after 7-day drought; and **(H)** the dry weight of root and shoot after 7-day drought. Values are the averages of 3 replicates ± SD. Different letters indicate significant differences according to Duncan’s multiple range tests (*P* < 0.05).

There was no significant difference between melatonin-treated and non-treated seedlings under the well-irrigated condition, and their growth was generally equivalent (Figure 1A). When we stopped watering and a progressive drought stress was applied, water content of soil in the pot decreased gradually (Figure 1C) and the growth of all seedlings was significantly inhibited, but the plants treated with melatonin had greener leaf tissues and higher chlorophyll contents than the non-treated plants (Figure 1A,F). Drought stress decreased both the length and the dry weight of shoots. Stopping watering increased the length of the longest root, but decreased the dry weight of all roots. And the application of exogenous melatonin could reverse these trends to some extents (Figure 1G,H). Consistently, the RWC (Figure 1D) and plant biomass of melatonin treated plants (Figure 1H) were significantly higher than those of the non-treated plants. And 100 μM melatonin could provide a better protection than 20 μM melatonin. Moreover, the root-irrigation method was more effective than the leaf spaying method, and the seedlings of root irrigation with 100 μM melatonin exhibited healthy growth with significantly higher chlorophyll contents and plant biomass. After rehydration, the turgidity of the leaf cells was regained and the slight withering leaves stood up again (Figure 1B). These results indicated that exogenous melatonin can help maize seedlings to resist the drought stress.

### The Effects of Exogenous Melatonin on Endogenous Melatonin Level

To evaluate the effect of drought stress on the melatonin biosynthesis, endogenous melatonin levels of the leaf tissues were quantified after the progressive drought stress for 7 days. The melatonin of the leaves was about 22 pg g^-1^ dry weight (DW) and the application of exogenous melatonin showed no remarkable induces of endogenous melatonin content under the well-irrigated condition (Figure 1E). When subjected to the drought stress, melatonin levels were markedly induced. Irrigation with 100 μM melatonin improved the level of endogenous melatonin under drought stress. These results suggested the melatonin is involved in the responses of maize seedlings to drought stress and the application of exogenous melatonin could change the accumulation of endogenous melatonin under stress.

### Exogenous Melatonin Treatment Decreased the Accumulation of ROS Under Drought Stress

**FIGURE 2:**
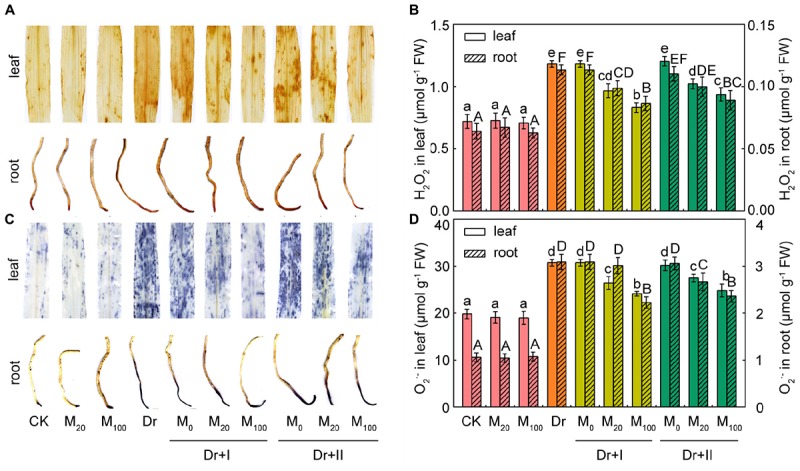
Reactive oxygen species accumulation in maize leaves and roots. **(A)** Histochemical detection of H_2_O_2_ level in leaf and root. **(B)** Quantification of H_2_O_2_ content in leaf and root. **(C)** Histochemical detection of O_2_^-^ level in leaf and root. **(D)** Quantifications of O_2_^-^ content in leaf and root. Values are the averages of 3 replicates ± SD. Different letters indicate significant differences according to Duncan’s multiple range tests (*P* < 0.05).

It has been reported that melatonin and its metabolites are highly effective ROS scavengers (Tan et al., 2007). The levels of two major ROS species, O_2_^-^ and H_2_O_2_, in leaves and roots were analyzed by histochemical staining and spectrophotometry. The application of exogenous melatonin led to no significant change under the well-irrigated condition. While significant H_2_O_2_ and O_2_^-^ bursts occurred in the leaves and roots under the drought condition, but exogenous melatonin application markedly reduced the accumulation of ROS (Figure 2). The melatonin treatment of 100 μM worked more effectively to scavenge ROS than 20 μM, and root-irrigation method was more efficient to slow the accumulation of ROS than the leaf spraying.

### Exogenous Melatonin Protected Cell Membranes and Alleviated Cell Death Under the Drought Stress

**FIGURE 3:**
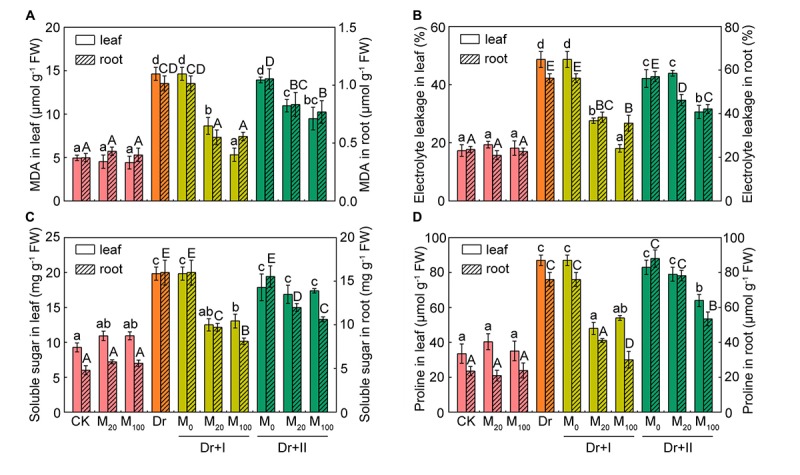
Membrane lipid peroxidation and osmolytes. The malondialdehyde (MDA) **(A)**, relative electrolyte leafage (EL) **(B)**, soluble sugar **(C)**, and proline **(D)** contents in leaf and root after 7-day drought stress. Values are the averages of 3 replicates ± SD. Different letters indicate significant differences according to Duncan’s multiple range tests (*P* < 0.05).

To evaluate the protective effect of exogenous melatonin on the integrity of membrane system under the drought condition, malonaldehyde (MDA) level and relative electrolyte leakage (EL) were investigated. MDA content and EL in the leaves and roots were not altered by exogenous melatonin under the normal growth condition (Figure 3A,B). MDA content and EL increased obviously in drought-stress seedlings, which suggested that drought damaged the integrity and fluidity of the cell membrane system. But exogenous melatonin markedly decreased the level of MDA and the rise of EL under the drought condition. At the same time, the root-irrigation method was more effective in protection of cell membrane than the leaf-spraying method. And the root irrigation with 100 μM melatonin was more effective in maintaining the stability of cell membrane and protecting from lipid peroxidation.

The leaf water potential often decreased during drought stress, and compatible solutes (such as proline and soluble sugar) could improve the cytoplasmic osmotic pressure, balance the water potential, protect the membrane system and the other biological macromolecules and thereby reduce water loss from cells. Under the well-irrigated condition, soluble sugar content of leaves increased slightly after the melatonin treatment, but there were no significant differences in both leaves and roots between the melatonin-treated and non-treated seedlings. Proline and soluble sugars increased under the drought stress in both leaves and roots. Root irrigation with melatonin could down-regulate levels of these osmotic substances under the drought stress. 20 and 100 μM melatonin treatments showed no statistical difference in proline and soluble sugar levels in leaves, but the roots of 100 μM melatonin treatment had lower levels of osmotic substances than those of 20 μM melatonin treatment after 7 days-drought stress. As for leaf spraying treatment, soluble sugar content of the leaves did not changed significantly, but markedly decreased in the roots (Figure 3C,D). Leaf spraying with 20 μM melatonin had no significant influence on proline level both in both leaves and roots, and 100 μM melatonin lowered their levels under the drought condition. These results suggested soluble sugar and proline in shoots and roots of the maize adopted different strategies to adapt to the progressive drought stress, and exogenous melatonin could partly reverse these changes. In general, the irrigation method exhibited a better protective effect than spraying.

**FIGURE 4:**
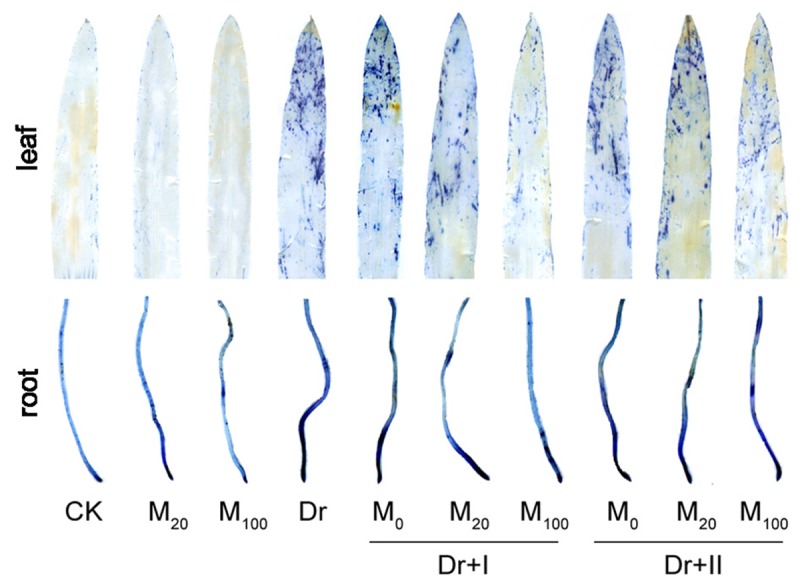
Effects of exogenous melatonin on cell death. The leaves and roots were stained by trypan blue after 7-day drought stress.

Cell death is usually induced by abiotic stresses. The tissues of leaves and roots showed obvious cell death under the drought condition, particularly in leaf tips and root tips (Figure 4). However, melatonin treatment alleviated the cell death, especially by the irrigation method of 100 μM melatonin.

### Exogenous Melatonin Protected Photosynthesis Under Drought Condition

**FIGURE 5:**
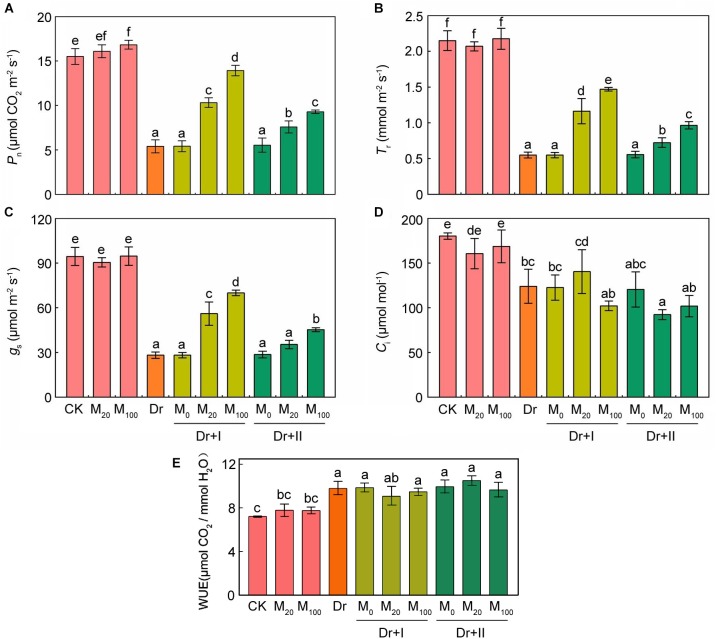
Effects of exogenous melatonin on photosynthetic parameters. **(A)** Net photosynthetic rate (Pn), **(B)** transpiration rate (Tr), **(C)** stomatal conductance (Gs), **(D)** intercellular CO_2_ concentration (C*i*), and **(E)** Water-use efficiency (WUE) after 7-days drought stress. Values are the averages of 3 replicates ± SD. Different letters indicate significant differences according to Duncan’s multiple range tests (*P* < 0.05).

Drought stress can inhibit photosynthesis. Under drought stress, melatonin treatment significantly inhibited the decrease in chlorophyll contents (Figure 1F). Gas exchange parameters were measured in order to explore the impacts of melatonin on photosynthesis. The application of exogenous melatonin did not significantly affect the gas exchange parameters under the well-irrigated condition. Net photosynthetic rate (*Pn*) (Figure 5A), transpiration rate (*Tr*) (Figure 5B), and stomatal conductance (*Gs*) (Figure 5C) all decreased under the water deficit condition, and the application of exogenous melatonin relieved these downtrends. Irrigation with 100 μM melatonin had a better protective effect. Intercellular CO_2_ concentration (*C_i_*) dropped under drought, whereas this decline was not reversed by the melatonin application (Figure 5D). These data showed that the CO_2_ assimilation significantly decreased under drought condition, and exogenous melatonin could provide a significantly protective role. Similar results have been demonstrated in cucumber (Zhang et al., 2013). Water-use efficiency (WUE; calculated as the ratio of net photosynthetic rate to transpiration rate) increased under the drought stress, however, kept stable after the exogenous melatonin treatments (Figure 5E).

**FIGURE 6:**
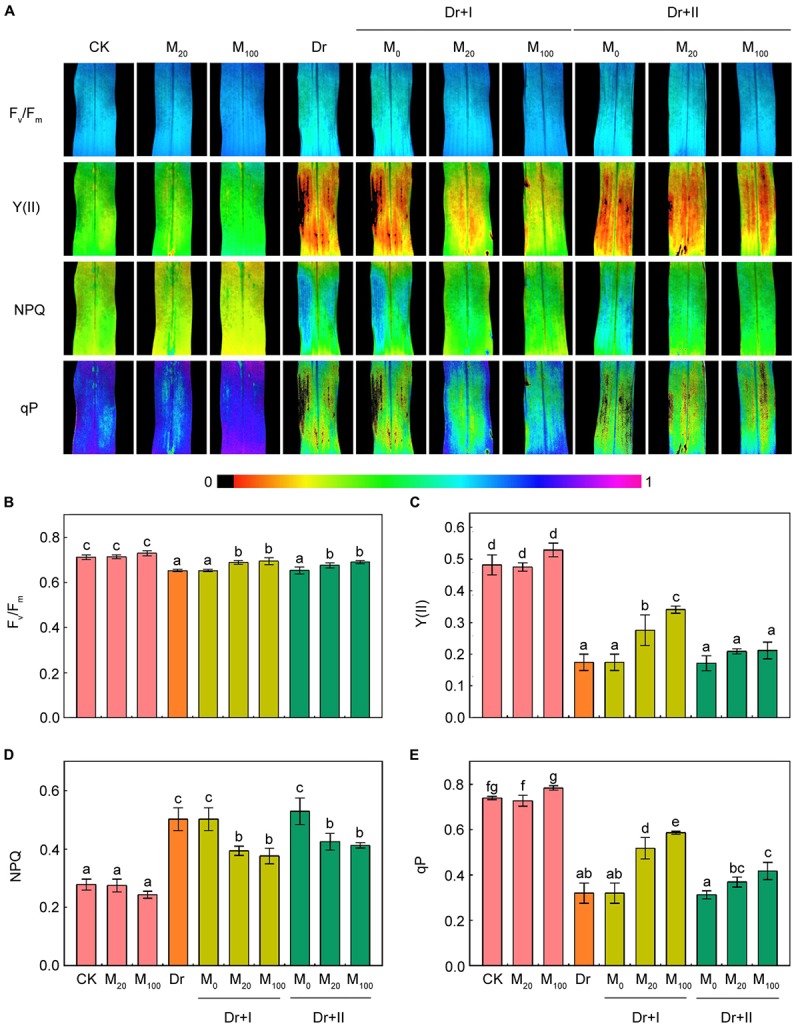
Effects of exogenous melatonin on chlorophyll fluorescence parameters. Quantitative values of maximum PSII yield (F_v_/F_m_) **(B)**, effective quantum yield of PSII [Y(II)] **(C)**, non-photochemical quenching (NPQ) **(D)**, and photochemical quenching (qP) **(E)** were matched with the fluorescence images **(A)**. Values are the averages of 3 replicates ± SD. Different letters indicate significant differences according to Duncan’s multiple range tests (*P* < 0.05).

Chlorophyll fluorescence is an effective tool to explore the work status of photosystem II (Wang et al., 2013a). The fluorescence images showed that under the well-irrigated condition, the application of melatonin did not change F_v_/F_m_, Y(II), NPQ and qP (Figure 6). F_v_/F_m_, Y(II) and qP decreased significantly under the drought condition. In contrast, the NPQ value rose, indicating that the thermal energy dissipation in PSII was enhanced. However, F_v_/F_m_, Y(II) and qP was increased and NPQ was decreased by exogenous melatonin treatments in maize seedlings under the drought stress. Meanwhile, the root-irrigation method worked more effectively than the leaf spraying method.

### The Effects of Exogenous Melatonin on Antioxidant System

Plants evolve antioxidant defense system to avoid the damages of ROS accumulation and lipid peroxidation. Thus, the changes of non-enzymatic antioxidants (AsA-DHA and GSH-GSSG) and enzymatic antioxidants (POD, CAT, SOD, APX, GPX, GR, and DHAR) of maize leaves and roots in the absence and presence of melatonin were determined.

**FIGURE 7:**
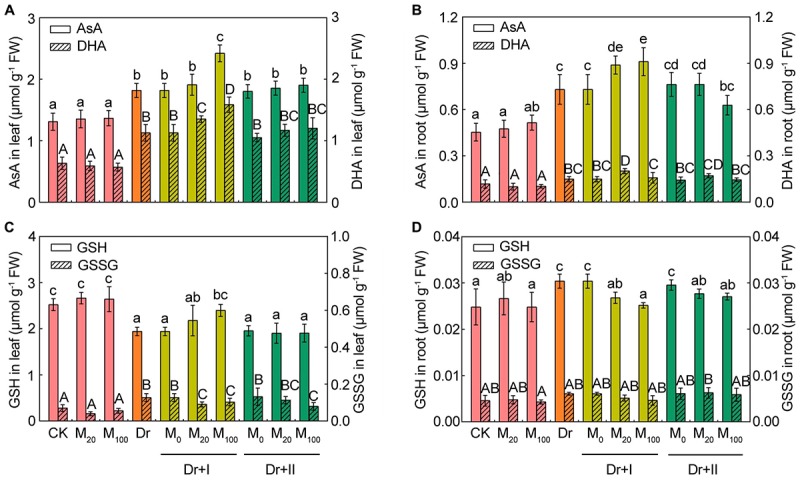
Effects of exogenous melatonin on the non-enzymatic antioxidants. The contents of reduced ascorbic acid (AsA) and dehydroascorbate (DHA) in leaves **(A)** and roots **(B)**, reduced glutathione (GSH) and oxidized glutathione (GSSG) in leaves **(C)** and roots **(D)** after 7-days drought stress. Values are the averages of 3 replicates ± SD. Different letters indicate significant differences according to Duncan’s multiple range tests (*P* < 0.05).

As shown in Figure 7, reduced AsA contents (Figure 7A) and DHA contents (Figure 7B) in leaves and roots tended to vary analogically. Under the drought condition, the contents of AsA and DHA significantly increased in both leaves and roots, and root irrigation with 20 μM melatonin further boosted AsA and DHA levels. 100 μM melatonin could further increase their levels in leaves, but there was no significant change for AsA and DHA in roots. Leaf-spraying with melatonin did not significantly change AsA and DHA contents in both leaves and roots, except that 100 μM melatonin decreased their levels in roots. Under the drought condition, the GSH level decreased in leaves but increased in roots, while the content of GSSG increased in leaves and almost remained unchanged in roots. Root irrigation with melatonin could improve the level of GSH and lower the GSSG content, but leaf spraying had no influence on GSH and GSSG in leaves (Figure 7C). However, both root irrigation and leaf spraying lowered the level of GSH and the GSSG remained unchanged in roots (Figure 7D). Overall, the melatonin application further increased AsA and GSH contents when exposed to the drought condition, and the root-irrigation method showed a better inductive effect than the leaf spraying method.

**FIGURE 8:**
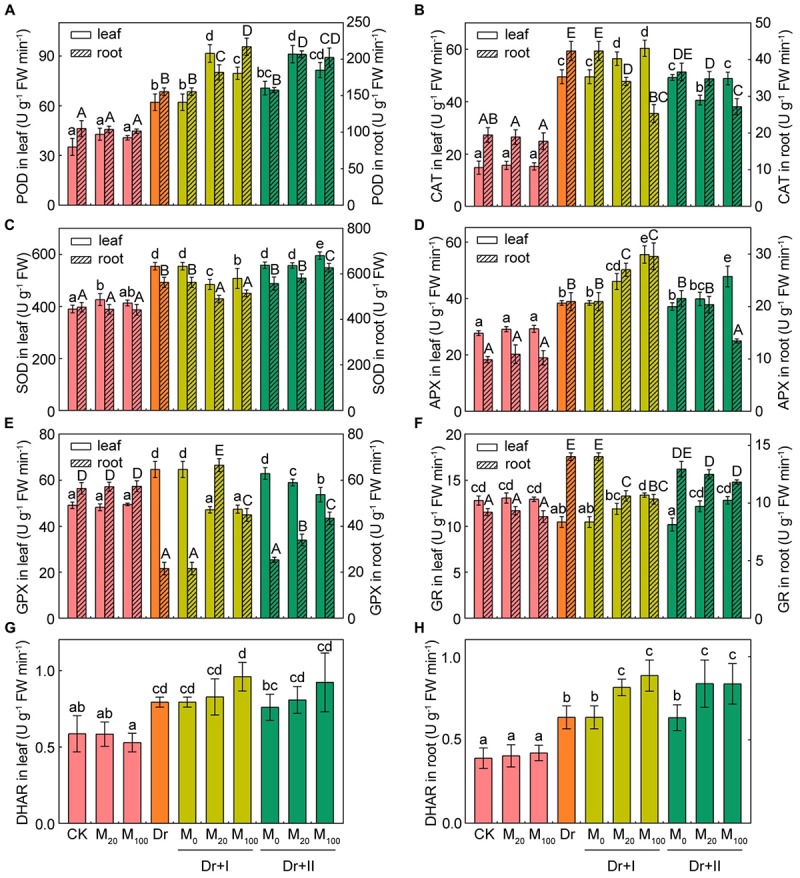
Effects of exogenous melatonin on antioxidant enzymes activities. The peroxidase (POD) **(A)**, catalase (CAT) **(B)**, superoxide dismutase (SOD) **(C)**, ascorbate peroxidase (APX) **(D)**, glutathione peroxidase (GPX) **(E)**, glutathione reductase (GR) **(F)**, and dehydroascorbate reductase (DHAR) **(G,H)** activities in leaves and roots after 7-days drought stress. Values are the averages of 3 replicates ± SD. Different letters indicate significant differences according to Duncan’s multiple range tests (*P* < 0.05).

The activities of the six antioxidant enzymes were hardly affected by exogenous melatonin under the well-irrigated condition. While the drought stress improved the antioxidant enzymic activities in both leaves and roots except the activities of GPX and GR. The activity of GPX reduced in roots and increased in leaves, but the activity of GR decreased in leaves but up-regulated in roots (Figure 8). The POD and DHAR activities increased in both leaves and roots no matter with root irrigation or leaf spraying (Figure 8A,G,H). Melatonin enhanced CAT activity in both leaves and roots, but leaf spraying method showed a weaker effect than the root-irrigation method (Figure 8B). Root irrigation with melatonin lowered SOD activity, but leaf-spraying with melatonin increased the activity in both roots and leaves (Figure 8C). The activity of APX was enhanced by the root-irrigation method in both leaves and roots, and the leaf-spraying method elevated its activity in leaves but reduced its activity in roots (Figure 8D). The activity of GPX reduced in leaves and increased in roots no matter with root irrigation or leaf spraying under the drought condition (Figure 8E). The activity of GR increased in leaves and decreased in roots no matter with root irrigation or leaf spraying under the drought condition (Figure 8F). These data indicated that antioxidant enzymatic activities made organ-specific responses to the drought stress. Yang et al. (2015) also found antioxidant enzymatic activities had tissue-specific responses to water logging. Furthermore, the effects of exogenous melatonin on the antioxidant enzymatic activities varied with the dosage, application methods and plant organs. Drought stress could induce distinct changes of antioxidant enzymatic activities in roots and leaves, and exogenous melatonin could weaken or strengthen these fluctuation trends.

### Melatonin Protected PSII by Increasing D1 Protein Level Under Drought Stress

**FIGURE 9:**
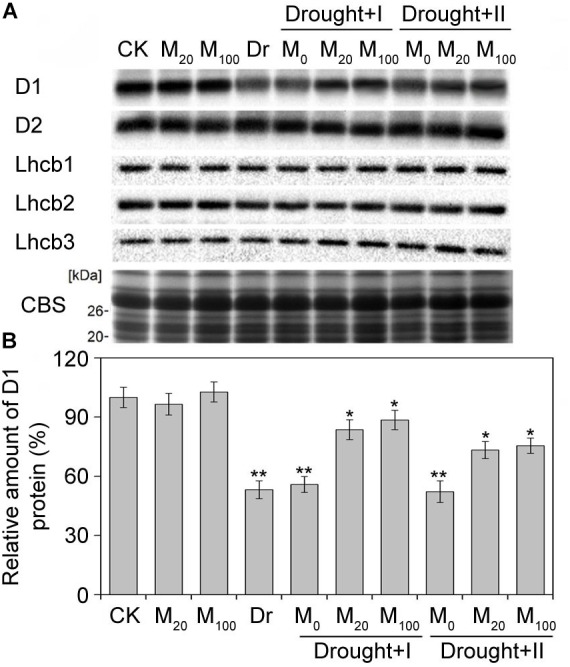
Effects of exogenous melatonin on thylakoid membrane proteins. Immunoblot analyses of thylakoid membrane proteins were performed with antibodies against D1, D2, Lhcb1, Lhcb2, and Lhcb3 **(A)**. Protein contained an equal amount of chlorophyll was loaded. SDS-PAGE stained with blue (CBS) are used as control. The quantification of D1 were shown at **(B)**. Values are the averages of 3 replicates ± SD. Results are relative to the amount of CK (100%). ^∗^, ^∗∗^ indicate statistically significant differences at *P* < 0.05 and *P* < 0.01, respectively, determined with Student’s *t*-test.

To gain insights into the photosystem II proteins changes by exogenous melatonin treatment under the drought stress, thylakoid membrane protein was analyzed by the immunoblotting (Supplementary Figures S1–S6). Melatonin treatment did not change the level of PSII protein under the well-irrigated condition. When exposed to the drought stress, D1 protein reduced significantly while the other proteins of PSII showed no significant changes (Figure 9). The melatonin treatment alleviated the decrease in D1 protein under the drought condition. The root-irrigation method showed a better protective effect on D1 protein than the leaf spraying method, and 100 μM melatonin could better protect D1 from damages than 20 μM concentration. These results indicated that melatonin application could alleviate the damages of PSII protein to maintain a higher PSII activity and normal photosynthesis under the drought stress.

## Discussion

It is well known that the growth, development and production of crops are inhibited by abiotic stresses (Rahdari and Hoseini, 2012). However, higher plants have evolved diverse physiological, biochemical and morphological strategies to response to drought (Farooq et al., 2009). Melatonin is a new plant growth regulator, and it has been reported to alleviate the oxidative damages caused by drought stress (Wang et al., 2013b; Zhang et al., 2013; Shi et al., 2015a; Wei et al., 2015; Ye et al., 2016). In the present experiment, the protective effects of two different melatonin-application methods in maize roots and leaves exposed to drought were investigated.

It has been reported that melatonin might play a key role in the regulation of plant growth and development (Tan et al., 2012; Wei et al., 2015). In this study, our results indicated that melatonin pre-treated plants presented higher chlorophyll levels, shoot height, and plant weight compared with the non-treated plants under the drought condition. These results were in agreement with the previous reports (Liu et al., 2015; Ye et al., 2016). Moreover, the melatonin pre-treated seedlings showed a higher level of endogenous melatonin under the drought stress, suggesting that the endogenous melatonin might be closely related to the drought resistance. In addition, we found that the root-irrigation method was more effective in improving drought resistance than the leaf spraying method.

It has been shown that the drought-induced stomatal closure could lead to the decline in photosynthetic capacity (Farooq et al., 2009). Here, we showed that drought stress declined the *Pn, Tr, Gs*, and *Ci*, while these decreases were partly recovered by exogenous melatonin application, except *Ci* (Figure 5). These results suggested that the application of exogenous melatonin might affect the stomata open status under drought stress. Similar research results were observed in tomato and apple (Wang et al., 2013b; Liu et al., 2015). In addition, our results further showed that root irrigation with melatonin provided better protective effects on the gas exchange than the leaf-spraying. Indeed, stomatal opening under drought can be also risky for plants because of the water loss through transpiration. However, carbon assimilation (photosynthesis) and transpiration rate increased simultaneously and thus the water-use efficiency (WUE) kept stable after the melatonin treatments (Figure 5E). Excessive stomatal opening under the drought stress may not occur after the melatonin treatments.

Chloroplast is the major source of free radical generation in plants, which require strong protection from free radicals and associated oxidative stress. It has been found that the biosynthesis of melatonin in plants might take place in chloroplast (Zheng et al., 2017). Previous studies showed that application of exogenous melatonin could induce endogenous melatonin biosynthesis in chloroplast (Zheng et al., 2017). In this study, we found that exogenous melatonin could change the accumulation of endogenous melatonin under drought stress and the increased endogenous melatonin might maintain chloroplast integrity and enhance net photosynthesis rate.

Chlorophyll fluorescence has become a powerful approach for the study of plant photosynthetic characteristics under various environmental stresses (Govindjee., 2004). Some studies showed that severe or long-time drought leads to photoinhibition in the PSII reaction center (Sperdouli and Moustakas, 2012; Chen et al., 2016b). In consistent with these findings, we found that Fv/Fm, ΦPSII, and qP significantly decreased under the drought stress (Figure 6). The reduction in Fv/Fm, qP and ΦPSII, and the increase in the level of NPQ suggested that drought stress induced a severe damage to photosynthetic apparatus in maize seedlings. In addition, it is well known that melatonin could improve the photosynthetic efficiency in higher plants under stressful conditions (Yin et al., 2013; Zhao et al., 2015; Jiang et al., 2016). In the present experiments, melatonin application significantly improved the photosynthetic capacity of PSII under the drought condition. These results were in accordance with previous studies in apple and cucumber (Wang et al., 2013a; Zhang et al., 2013). In addition, higher photochemical capacity was observed for the root-irrigation method, suggesting that the applications to the roots were more efficient compared with leaves as far as the protective roles of exogenous melatonin to the photosynthetic efficiency under environmental stresses were concerned.

Photosystem II has been known as a primary target of photodamages under environmental stresses (Kato et al., 2012). When the PSII photodamage rate exceeds the repair ability, photoinhibition becomes apparent (Nishiyama et al., 2006). However, *de novo* synthesis of D1 protein is necessary for the repair cycle of PSII. It has been reported that drought stress inhibits the protein synthesis of D1 (Chen et al., 2016b). In accordance with the previous report, our work also showed that D1 protein decreased obviously under the drought stress. However, melatonin has been reported to protect PSII proteins from oxidative injuries and regulate the levels of senescence-associated proteins (Byeon et al., 2012; Zhang et al., 2015). A recent research indicated that melatonin is effective in the process of the PSII repair by maintaining the protein availability of D1 in tomato under salt (Zhou et al., 2016). Our work further confirmed the protective role of melatonin on PSII proteins in maize under drought stress. Melatonin with two different applications partly counteracted the decline in D1 protein under the stress. Therefore, these results suggested that melatonin played a vital role in maintaining photosynthetic efficiency by regulating the repair cycle of PSII under environmental stresses.

Under stress conditions, plants usually generate much ROS, which subsequently induce the peroxidation of membrane lipids and oxidative damages (Munné-Bosch and Peñuelas, 2003; Kar, 2011). However, previous studies had shown that melatonin played an important role in the detoxification of reactive oxygen and free radicals and functions as an antioxidant in living organisms (Reiter, 1998; Tan et al., 2000a; Reiter et al., 2007). As a broad-spectrum antioxidant, melatonin can directly eliminated ROS and the subsequent products, its derivatives, AFMK (N1-acetyl-N2-formyl-5-methoxyknuramine) and AMK (N1-acetyl-5-methoxykynuramine), can also scavenge ROS, and further terminate the cascade reaction of lipid peroxidation (Ressmeyer et al., 2003; López-Burillo et al., 2010). Thus, one molecule of melatonin may eventually scavenge ten molecules of radicals at least (Tan et al., 2007). Melatonin treatment can markedly decreased the content of ROS and thus alleviate oxidative damages induced by the excessive ROS accumulation (Wang et al., 2013b; Meng et al., 2014; Shi et al., 2015a,c). Our results showed that drought induced the significant accumulation of ROS in the maize leaves as well as higher levels of EL and MDA, which are important oxidative-damage indicators of the integrity of cell membranes (Liu et al., 2015). However, exogenous melatonin treatment obviously alleviated oxidative damages of leaves, especially with the root-irrigation method, suggesting that melatonin application might effectively protect cell membranes against oxidative damages under drought stress. Melatonin is a lipophilic and hydrophilic molecule and can distribute in cytoplasm and lipid membranes (Angel, 2007). Melatonin, located in hydrophilic side of the lipid bilayer, prevents biological membrane from the lipid peroxidation by directly neutralizing the toxic reactants (Ceraulo et al., 1999; de Lima et al., 2010). Interestingly, the organization of melatonin in lipid membranes depends on its concentration. At low concentrations, the melatonin molecules arrange parallel to the lipid tails; in contrast, they arrange parallel to the bilayers at high concentrations (Dies et al., 2015). The location of melatonin in the lipid bilayer is speculated to monitor disordering in the hydrophobic tail of lipid bilayer.

Drought stress often leads to the instability of cell membranes in plants (Wang and Huang, 2004). Osmotic regulation is thought to be the most important basic response to drought stress (Ali et al., 2013). Soluble sugars and proline, as two key osmotic regulators, often increase in plants under drought stress (Xiong and Zhu, 2002), which were also confirmed in the present work. However, melatonin application significantly decreased proline and soluble sugar levels, especially with the root-irrigation method. Therefore, our results indicate that melatonin may maintain a positive turgor pressure to meet the water balance.

In addition, ROS could increase the permeability of cell membranes and subsequently result in cell death (Alvarez et al., 1998). Our result of trypan-blue staining indicated that exogenous melatonin application can effectively reduce cell death by scavenging ROS.

In order to reduce stress-triggered ROS accumulation, plants have developed a complex array of enzymatic and non-enzymatic defense systems against oxidative damages (de Souza et al., 2014). Many studies have found that antioxidant enzymatic activities could respond to osmotic stress (Chen et al., 2017), and exogenous melatonin application could regulate some antioxidant enzymatic activities to alleviate the stress-induced ROS burst in plants (Tan et al., 2000b; Shi et al., 2015a). Our results indicated that melatonin application promoted some antioxidant enzymatic activities of maize roots and leaves under the drought condition, especially with the root-irrigation method. Previous studies showed that application of exogenous melatonin could induce endogenous nitric oxide generation (Zhao et al., 2018), which has emerged as an important signaling molecule in plants, activating ROS scavenging enzymes under drought conditions (Kolbert et al., 2005). Meanwhile, melatonin could decrease *miR398s* expression that could activate ROS scavenging enzyme gene expression, such as *Cu/Zn SOD* and *Mn SOD* (Gu et al., 2017). Thus, nitric oxide is required for melatonin-enhanced tolerance against abiotic stresses, which might down-regulate *miR398* expression to activate ROS scavenging enzymatic activities and promote the expression of related genes and finally scavenging intracellular ROS. Furthermore, the AsA-GSH cycle played an important role against oxidative damages in plants (Zhang et al., 2015). Melatonin could act as a bridge to contact water-soluble antioxidants (e.g., AsA, GSH, and NADPH) with lipid-soluble antioxidants (e.g., Ve) to forms an antioxidant network (Mahal et al., 1999; Tan et al., 2005). Previous studies showed that melatonin application could maintain higher levels of GSH and AsA (Wang et al., 2013a; Shi et al., 2015a). Consistent with these reports, our study suggested that AsA and GSH contents of leaves were markedly induced in melatonin-treated seedlings under the drought stress, although melatonin application decreased the GSH content of roots. These results suggested that melatonin can maintain tissular redox homeostasis through activating the antioxidative defense system and subsequently improve the drought resistance of maize seedlings. Yang et al. (2015) reported that the plants of the genus Plantago showed organ-specific responses to submergence stress at the level of ROS, non-enzymatic antioxidants and the activities of antioxidative enzymes. We found the leaves and roots of maize seedlings showed different responses to the drought stress.

The multicellular plant is an organic whole, and all the organs in plant are interrelated. Plants usually face with numerous environmental stresses during their growth, and the adaption to the varying circumstances in different organs or tissues of plant is not independent. The tissues of plants can respond to environmental stresses far away from the primary attacking organs, and these strategies are called “systemic acquired acclimation” (SAA) ( Rossel et al., 2007; Burns et al., 2018). Our results indicated that the signal of exogenous melatonin could be transmitted across organs in plants. under drought stress, the exogenous melatonin applied to the roots could not only reduce the accumulation of ROS, affect antioxidative enzyme activities in roots, but also promote growth of the seedlings and improve photosynthesis in leaves. At the same time, the exogenous melatonin applied to the leaves not only reduced cell death and improved photosynthesis efficiency in leaves, but also affected the growth and metabolic activities in roots under drought stress.

In summary, the protective roles of melatonin with two different application methods to drought stress in both maize roots and leaves were investigated by comparing the antioxidative defense system, ROS accumulation levels and photosynthetic characteristics. Our results further demonstrated that the application of exogenous melatonin can alleviate the drought-induced damages and improve drought tolerance in plants through the activation of antioxidative defense systems and the elimination of ROS. Moreover, the melatonin application with the root irrigation showed more effective protective roles than the leaf-spaying method, and the signal of exogenous melatonin could be transmitted across organs in the plant.

## Data Availability

All datasets for this study are included in the manuscript and the Supplementary Files.

## Author Contributions

SY, MY, and Y-EC conceived the study. BH, Y-QZ, C-BD, J-QL, CH, L-JZ, and Z-WZ performed the experiments and carried out the analysis. BH, MY, and Y-EC wrote the manuscript.

## Conflict of Interest Statement

The authors declare that the research was conducted in the absence of any commercial or financial relationships that could be construed as a potential conflict of interest.

## Supplementary Material

FIGURE S1 Effect of exogenous melatonin on D1 under drought stress.

FIGURE S2 Effect of exogenous melatonin on D2 under drought stress.

FIGURE S3 Effect of exogenous melatonin on Lhcb1 under drought stress.

FIGURE S4 Effect of exogenous melatonin on Lhcb2 under drought stress.

FIGURE S5 Effect of exogenous melatonin on Lhcb3 under drought stress.

FIGURE S6 Isolation of thylakoid membranes proteins by SDS-PAGE. Protein contained an equal amount of chlorophyll was loaded. SDS-PAGE stained with blue (CBS) are used as control.
