# Supplementary figures and images for: Exogenous Melatonin Alleviates Oxidative Damages and Protects Photosystem II in Maize Seedlings Under Drought Stress
Source: Front Plant Sci. 2019 May 24;10:677. doi: 10.3389/fpls.2019.00677 (PMC6543012; doi:10.3389/fpls.2019.00677)

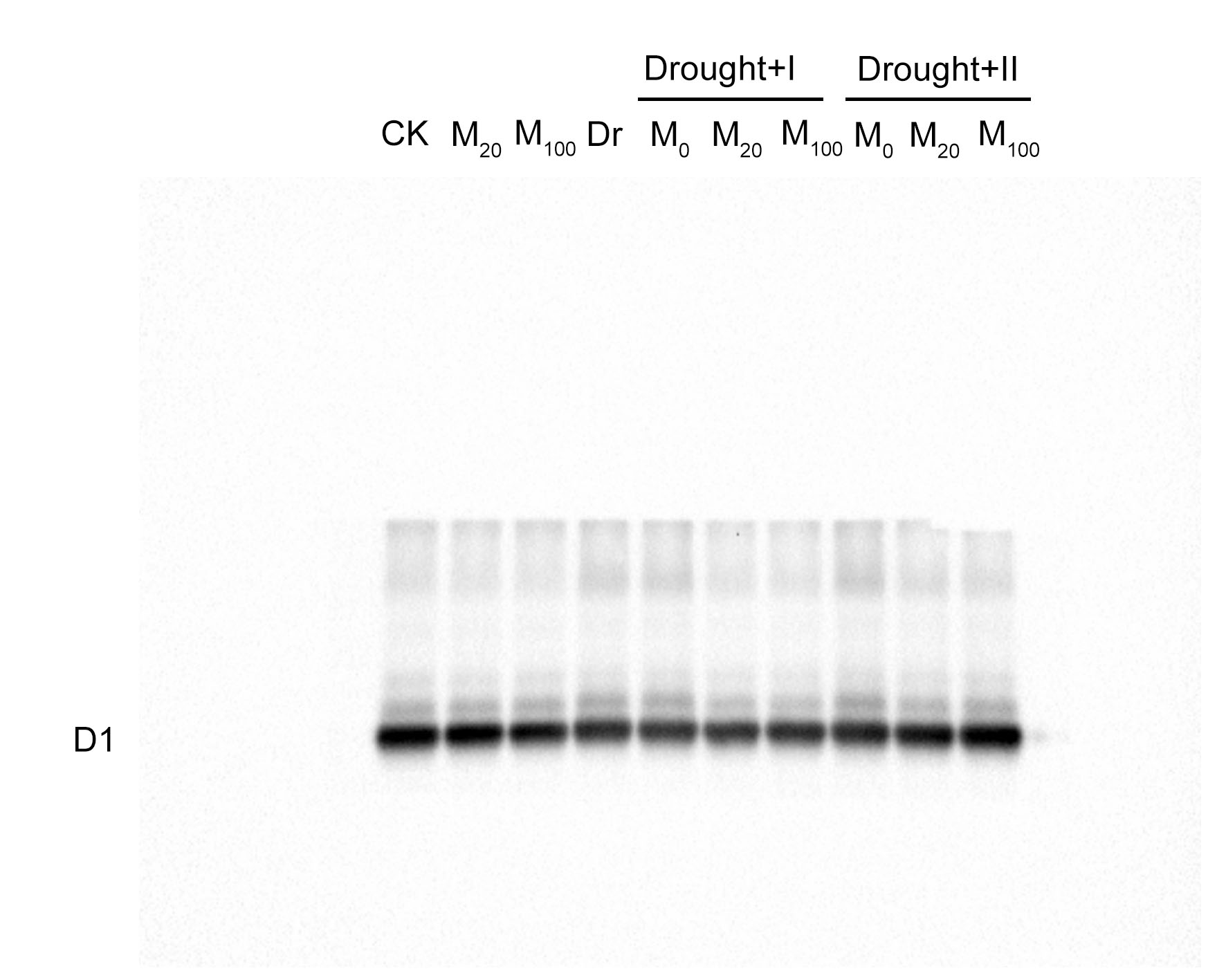

Supplement: FIGURE S1 — Effect of exogenous melatonin on D1 under drought stress. [file Data_Sheet_1.ZIP › Supplementary Material/Figure S1.jpg]

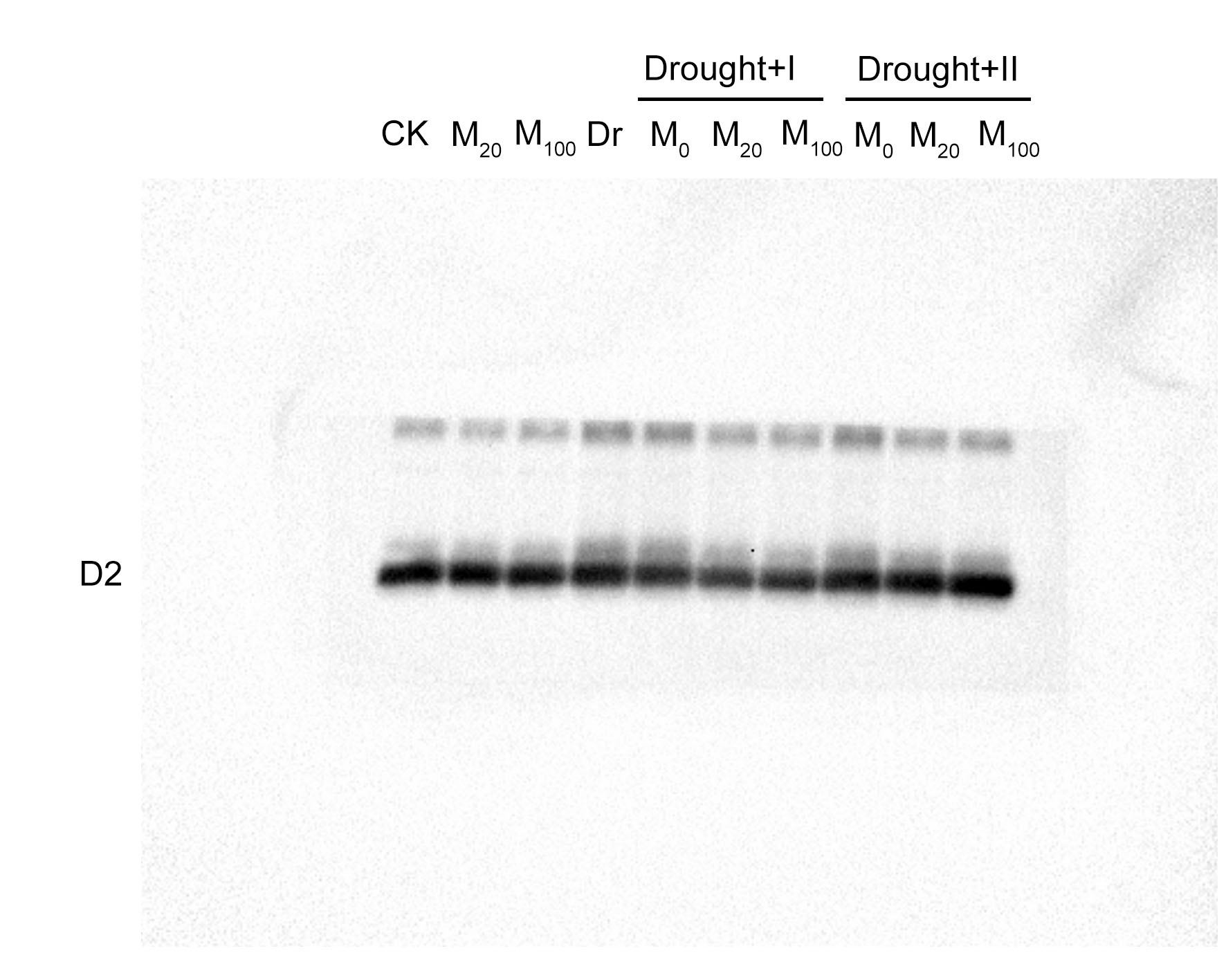

Supplement: FIGURE S1 — Effect of exogenous melatonin on D1 under drought stress. [file Data_Sheet_1.ZIP › Supplementary Material/Figure S2.jpg]

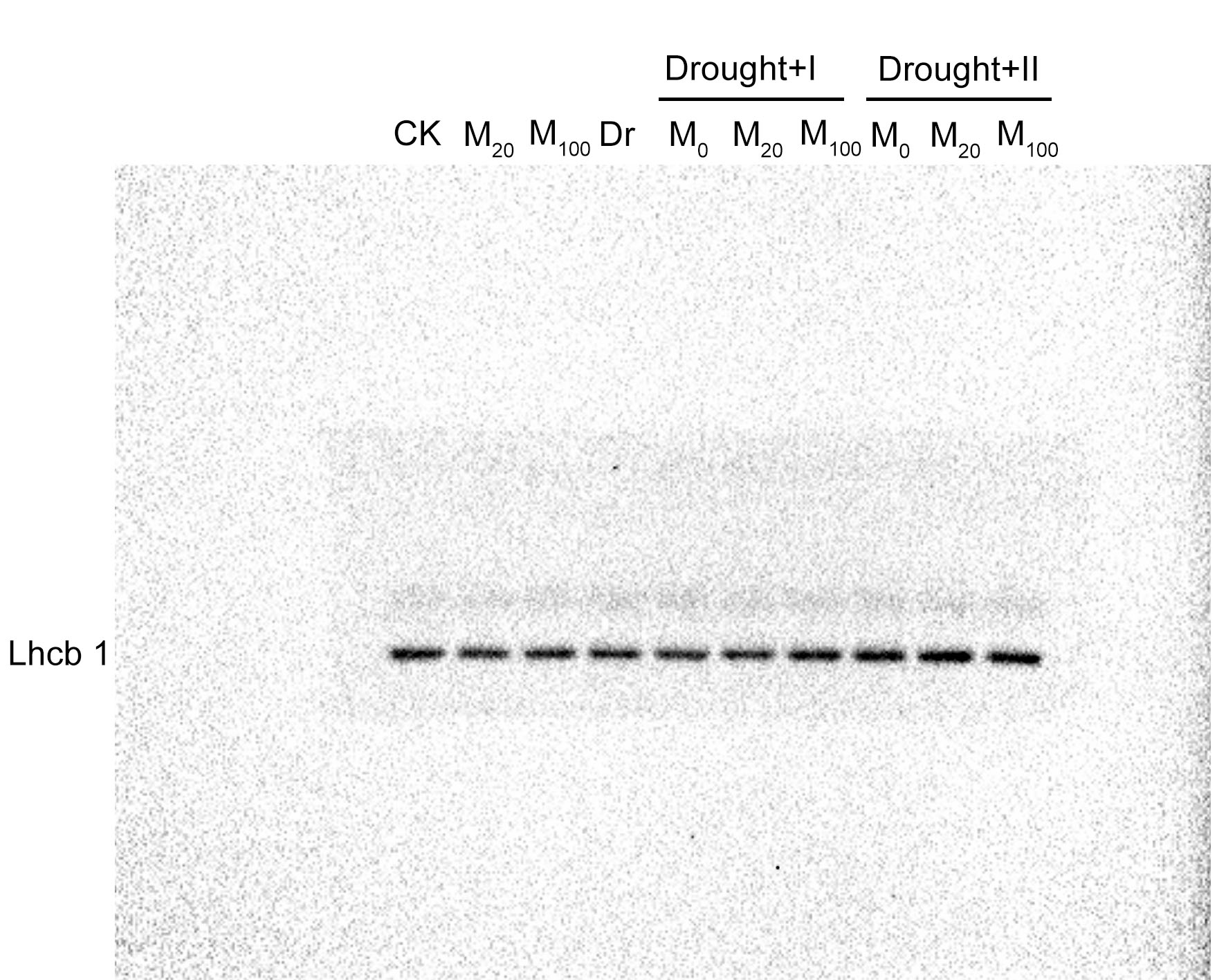

Supplement: FIGURE S1 — Effect of exogenous melatonin on D1 under drought stress. [file Data_Sheet_1.ZIP › Supplementary Material/Figure S3.jpg]

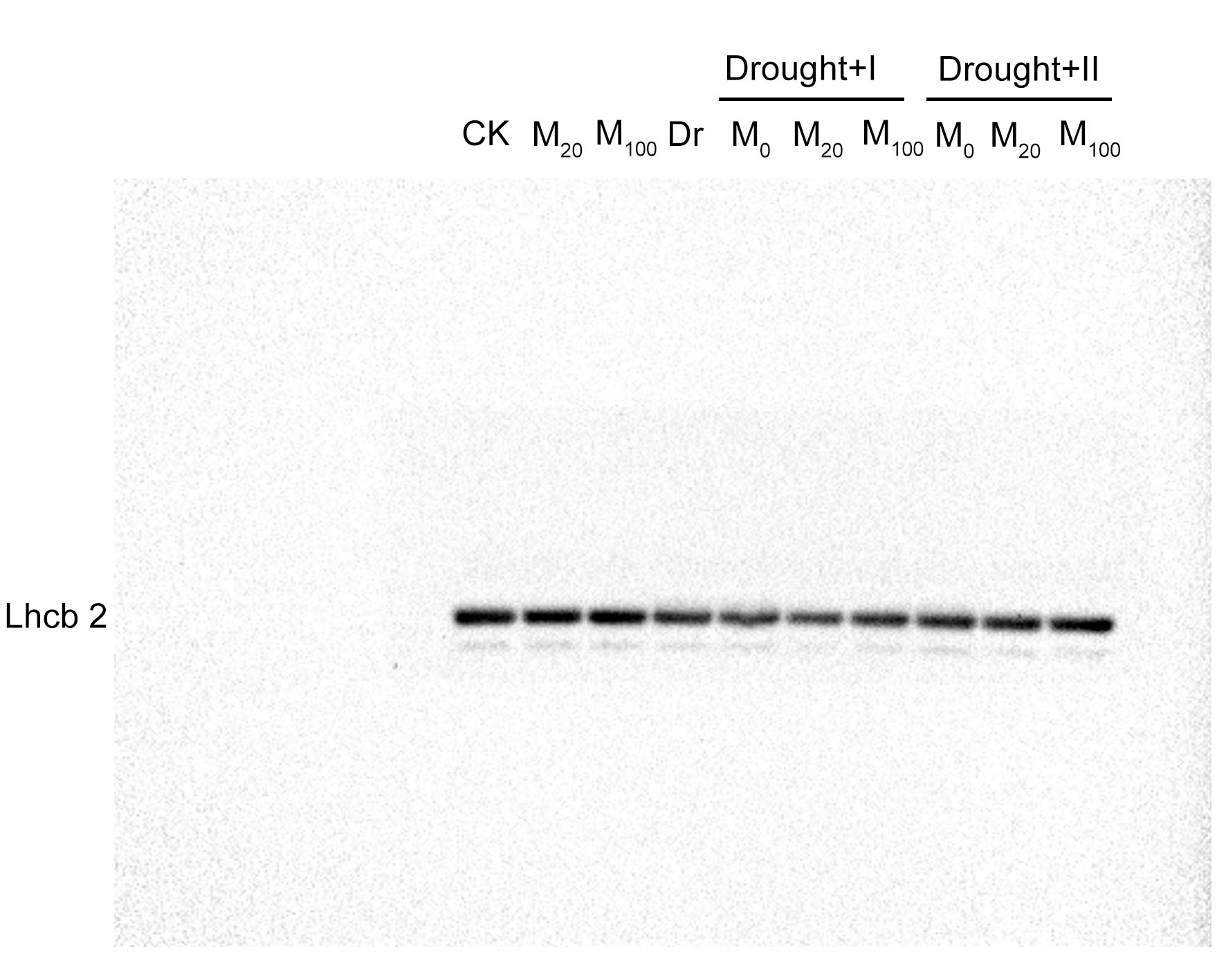

Supplement: FIGURE S1 — Effect of exogenous melatonin on D1 under drought stress. [file Data_Sheet_1.ZIP › Supplementary Material/Figure S4.jpg]

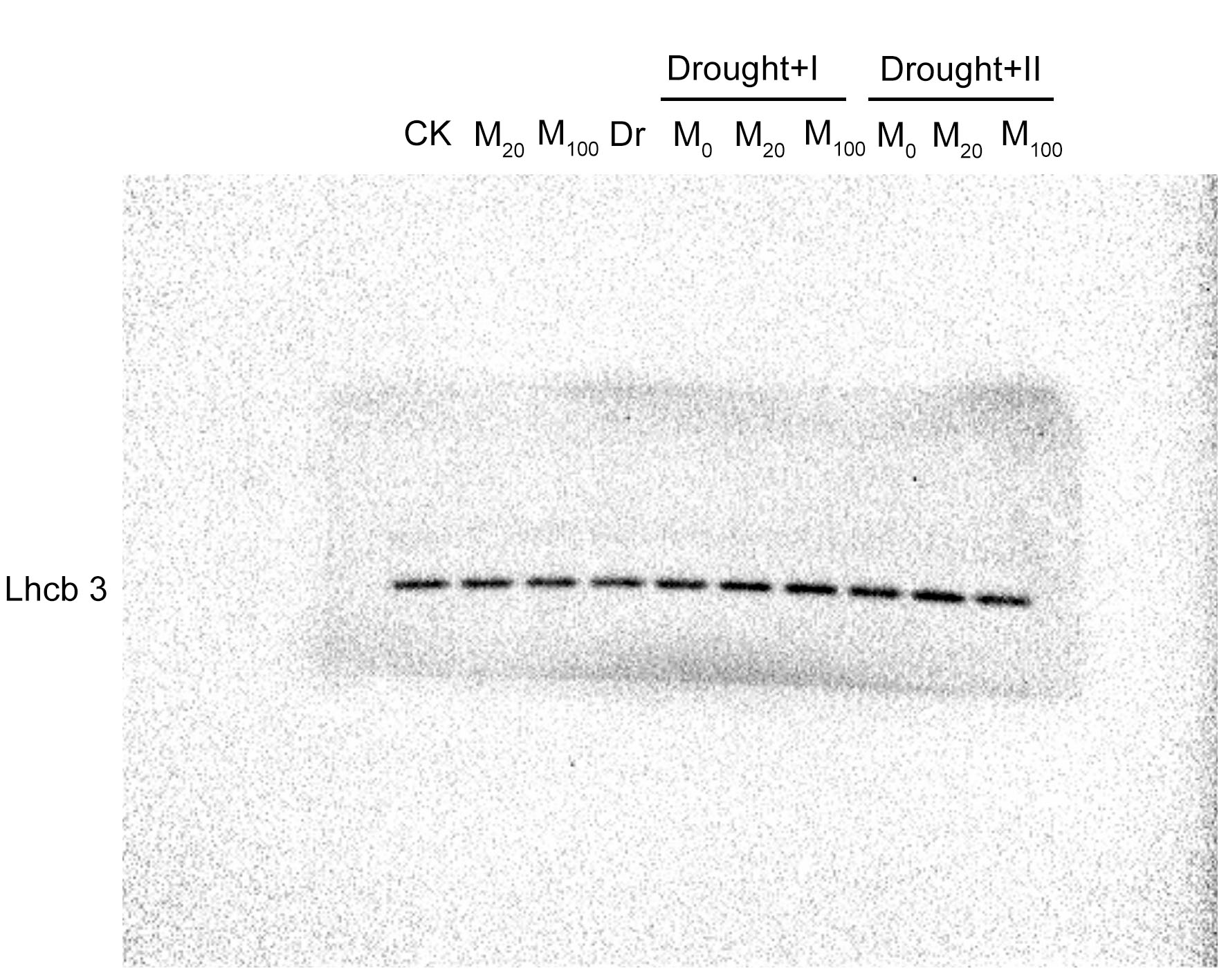

Supplement: FIGURE S1 — Effect of exogenous melatonin on D1 under drought stress. [file Data_Sheet_1.ZIP › Supplementary Material/Figure S5.jpg]

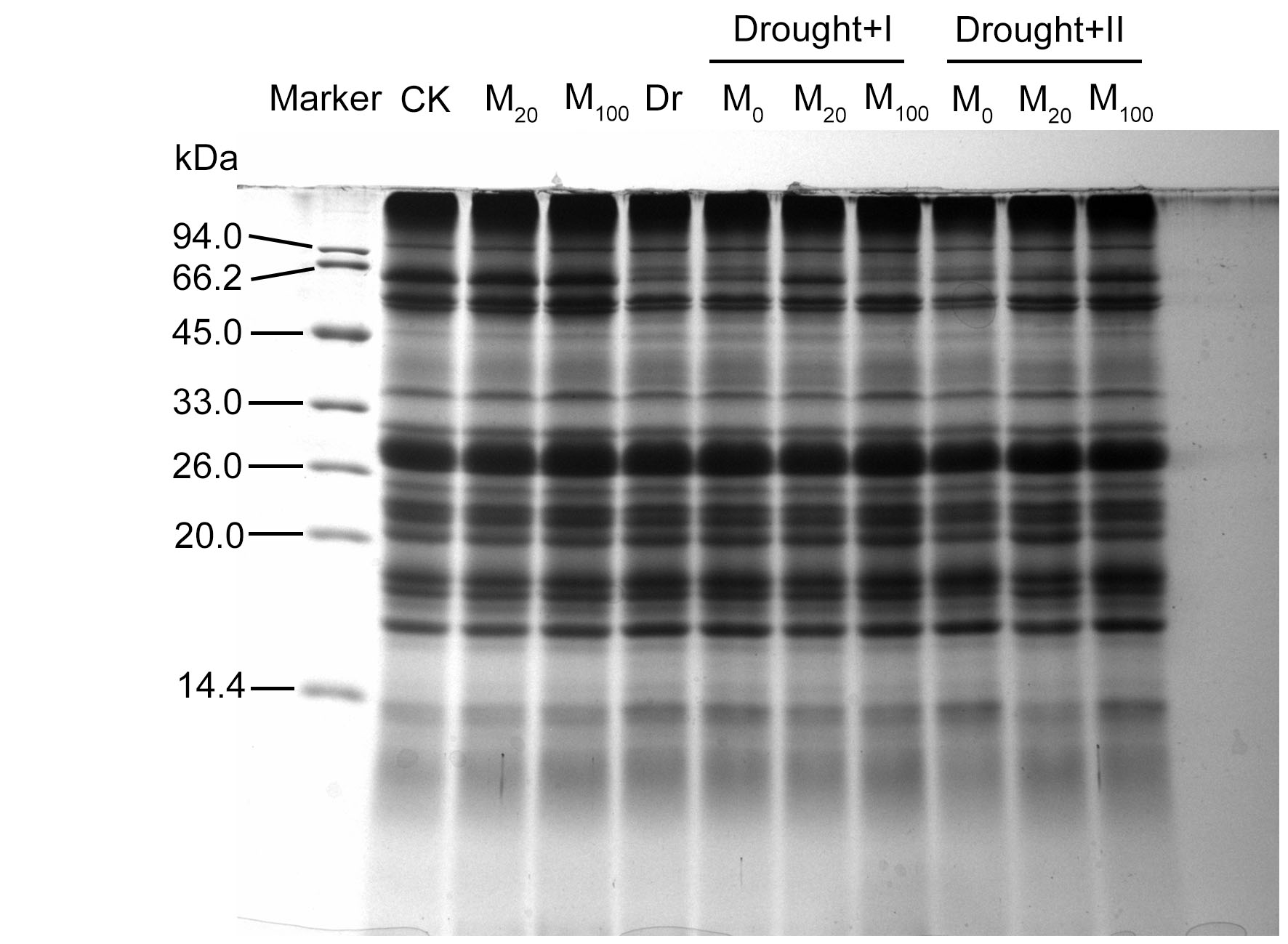

Supplement: FIGURE S1 — Effect of exogenous melatonin on D1 under drought stress. [file Data_Sheet_1.ZIP › Supplementary Material/Figure S6.jpg]
